# A predictive model for metabolic syndrome in a community-based population with sleep apnea: a secondary prevention screening tool using simple and accessible indicators

**DOI:** 10.3389/fnut.2025.1667055

**Published:** 2025-11-05

**Authors:** Tong Feng, Qiong Ou, Guangliang Shan, Yaoda Hu, Huijing He

**Affiliations:** ^1^Department of Respiratory and Critical Care Medicine, Deyang People's Hospital, Deyang, China; ^2^Sleep Center, Department of Respiratory and Critical Care Medicine, Guangdong Provincial People's Hospital (Guangdong Academy of Medical Sciences), Southern Medical University, Guangzhou, China; ^3^Department of Epidemiology & Biostatistics, Institute of Basic Medical Sciences, Chinese Academy of Medical Sciences/School of Basic Medicine, Peking Union Medical College, Beijing, China

**Keywords:** obstructive sleep apnea, metabolic syndrome, predictive models, community population, SHAP (SHapley Additive exPlanations)

## Abstract

**Objective:**

To establish a secondary prevention screening model for predicting metabolic syndrome (MetS) based on community obstructive sleep apnea (OSA) screening, using simple and easily accessible indicators, to help early identification of high-risk individuals and improve prognosis and reduce mortality.

**Methods:**

This study enrolled adults newly diagnosed with OSA from community settings in China, collecting comprehensive demographic and lifestyle data. To identify key predictive variables, least absolute shrinkage and selection operator (LASSO) regression was employed for feature selection. Nine machine learning algorithms, such as logistic regression, random forest, and support vector machine (SVM), were then used to build predictive models, with each undergoing rigorous training, hyperparameter tuning, and evaluation on stratified training, validation, and test datasets. Model performance was evaluated using multiple metrics, including the area under the receiver operating characteristic curve (AUC-ROC), accuracy, sensitivity, specificity, F1 score, calibration curves, and clinical decision curve analysis (DCA). To improve interpretability, Shapley additive explanations (SHAP) analysis was applied to quantify each predictor's contribution to the model's output.

**Results:**

Among the nine machine learning algorithms evaluated, the logistic regression model exhibited superior performance. The finalized model achieved an AUC of 0.814 on the test dataset, demonstrating strong discriminative ability. Key performance metrics included a sensitivity of 0.794, specificity of 0.647, accuracy of 0.693, and an F1 score of 0.617. Feature importance analysis highlighted body mass index (BMI), age, and gender as the most significant predictors of MetS. Calibration curves and clinical DCA further confirmed the model's reliability, showing close alignment between predicted probabilities and observed outcomes, thus affirming its clinical utility. External validation reinforced the model's robustness, yielding an AUC of 0.818, with consistent discrimination and well-calibrated predictions.

**Conclusion:**

This study successfully developed a MetS prediction model based on community environment. The model relies solely on simple, easily obtainable self-reported indicators and demonstrates good predictive performance. This model, as a primary screening tool, enables residents to assess their MetS risk status independently, without relying on complex biochemical tests or the assistance of specialized medical personnel.

## Graphical Abstract

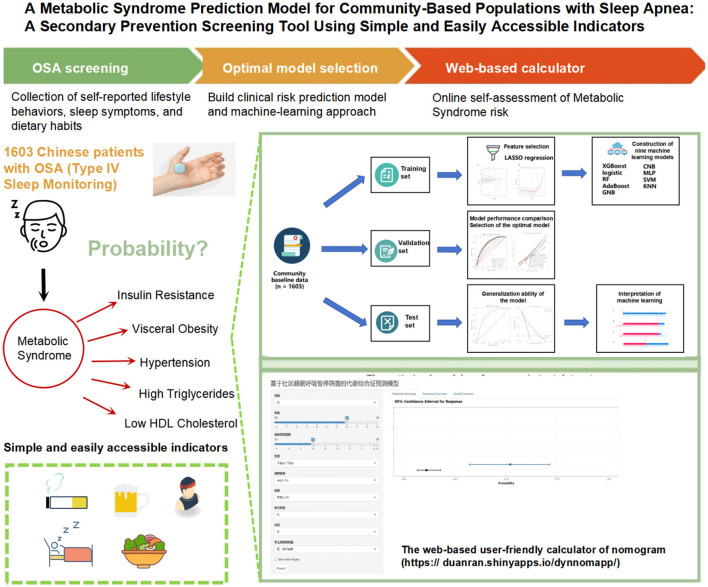

## 1 Introduction

Obstructive sleep apnea (OSA) and metabolic syndrome (MetS) pose significant global public health challenges. Recent epidemiological data estimate that approximately 936 million people worldwide are affected by OSA, establishing it as a pressing healthcare concern. China bears the highest burden, with around 176 million individuals diagnosed with the condition (1). Meanwhile, the prevalence of MetS among adults exceeds 20% globally (2), and this rate increases with age. These two conditions can form a vicious cycle, each exacerbating the other, thereby significantly elevating the risk of cardiovascular diseases, diabetes, and other chronic illnesses, and severely compromising patients' quality of life and health outcomes.

Our previous research focused on predicting health risks associated with OSA, through the development of a hypertension prediction model targeting individuals with OSA in community settings. This model was designed to identify cases of “masked hypertension”—hypertension that is not easily detectable during routine clinical visits but is closely linked to nocturnal hypoxic events (3). Hypertension is not only a common comorbidity of OSA but also a core diagnostic component of MetS. Early identification and integrated management of MetS have been shown to significantly reduce the incidence of cardiovascular diseases and related complications.

However, existing models for predicting MetS have notable limitations. These predictive models frequently integrate a diverse array of biochemical indicators, including key liver enzymes such as alanine aminotransferase (ALT) and aspartate aminotransferase (AST) (4, 5), alongside other indicators like blood glucose, blood pressure, lipid profiles, and waist circumference. The reliance on such complex combinations of parameters limits their practicality in clinical settings, as they typically require specialized laboratory testing, increasing both the financial and time burdens on patients. This in turn restricts their scalability for large-scale population screening. Therefore, there is an urgent need for simpler, more accessible predictive indicators that can facilitate MetS screening and early intervention in community settings.

Although some studies have investigated the long-term impact of OSA on MetS outcomes, a key challenge remains in effectively identifying high-risk individuals within communities to enable timely intervention and reduce mortality (6). To address this, the present study aims to develop a practical and effective MetS prediction model based on simple and user-friendly screening tools. To ensure feasibility, efficiency, and cost-effectiveness in real-world community contexts, the model will exclude biochemical markers and sleep stage data. Instead, it will utilize demographic and lifestyle information to identify predictive factors for MetS and construct a self-assessment model suitable for community use. This model is intended to improve the early detection of MetS and could be implemented via mobile applications or online surveys as a screening tool for secondary prevention. It may assist individuals in self-screening prior to consulting a community physician, or be employed by primary care providers during community-based OSA screening efforts.

## 2 Methods

### 2.1 Study population

The target population of this study was drawn from the “Chinese Academy of Medical Sciences Lifelong Health and Informatics Infrastructure Project (Guangdong Cohort),” implemented in Guangdong Province. Participants were recruited from the general community population between April 9 and May 18, 2021. The design and execution of baseline data collection in this cohort followed the methodological framework outlined in Data Resource Profile: The China National Health Survey (CNHS) (7).

A total of 3,829 individuals completed valid sleep monitoring, among whom 1,669 were identified with OSA. After excluding individuals without diagnostic data for MetS, a final sample of 1,603 diagnosed OSA patients meeting the study criteria was included in the analysis.

For predictive model development, it is generally recommended that the sample size should be 10 to 20 times the number of factors to be analyzed (8). In this study, approximately 21 variables are expected to be included in the model. Based on the upper limit of this recommendation (20 times), the minimum required number of outcome events is 21 × 20 = 420. Given that the reported incidence of the outcome is approximately 49% (9), the total sample size should be at least 420/49% = 857 participants.

### 2.2 Nocturnal sleep monitoring

In this study, we screened for OSA in a community-based population using a Type IV sleep monitoring device from Chengdu Yunweikang Health Technology Co., Ltd. (China). The device utilizes photoplethysmography (PPG) technology to measure pulse oxygen saturation (SpO_2_) by analyzing the differential absorption of hemoglobin under specific wavelengths of infrared and red light. To ensure data accuracy, the system incorporates advanced motion artifact detection algorithms, which effectively minimize signal interference caused by movement, thereby enhancing the reliability of monitoring durations and the validity of the collected physiological data (Supplementary Figure 1).

A comparative validation study was conducted to assess the diagnostic accuracy of a Type IV portable sleep monitoring device against the gold-standard polysomnography (PSG; Alice 6 model) at the Sleep Center of Guangdong Provincial People's Hospital (10). The study involved 196 participants, with OSA defined by an apnea-hypopnea index (AHI) of ≥5 events per hour. The Type IV device demonstrated robust diagnostic performance, achieving a sensitivity of 93%, specificity of 77%, and an area under the receiver operating characteristic curve (AUC) of 0.95, reflecting strong concordance with PSG results (Supplementary Figure 2).

A subgroup of 305 participants from Shantou City also underwent type III home sleep apnea testing (HSAT, model: Alice Night One) (11). The oxygen desaturation index (ODI) from the Type IV monitoring device showed a statistically significant correlation with the AHI measured by HSAT (*R*^2^ = 0.504, *P* < 0.001). Bland-Altman analysis further demonstrated that 93.1% (284/305) of paired measurements between the Type IV ODI and HSAT-derived AHI fell within the 95% limits of agreement, indicating strong clinical concordance between the two methods (Supplementary Figure 3).

Due to its simplicity, accessibility, and affordability, the reliability of type IV sleep monitoring in OSA screening has been validated across various populations, including individuals with obesity, surgical patients, people living with HIV, and patients with atrial fibrillation (12–15). When used alone or in combination with the STOP-Bang questionnaire, the type IV device significantly outperforms the STOP-Bang questionnaire alone in screening accuracy (16). While there may be some discrepancies in precise AHI estimation, the U.S. Preventive Services Task Force has stated that type IV sleep monitors generally offer high diagnostic accuracy for OSA (17).

Among participants who completed valid sleep monitoring, the prevalence of chronic obstructive pulmonary disease, asthma, and heart disease was below 5%. Moreover, all five study sites were located at altitudes below 500 m, minimizing the potential influence of altitude on oxygen saturation. The presence of OSA was defined based on an ODI (≥4% desaturation) threshold of ≥5 events per hour (13).

### 2.3 Definition of metabolic syndrome

According to the Guidelines for the Prevention and Treatment of Diabetes in China (2024 Edition), MetS is defined by the presence of at least three of the following components (18):

Hypertension: Systolic blood pressure ≥130 mmHg, diastolic blood pressure ≥85 mmHg, or current use of antihypertensive medications;

Elevated triglyceride levels (TG): ≥1.70 mmol/L;

Low high-density lipoprotein cholesterol (HDL-C): < 1.04 mmol/L;

Abdominal obesity: Waist circumference ≥85 cm in females or ≥90 cm in males;

Elevated fasting blood glucose: Fasting plasma glucose ≥6.1 mmol/L, or current use of antidiabetic medications.

### 2.4 Selection of candidate predictive variables

Relevant variables for predicting MetS were extracted from the survey questionnaire. These variables are all self-reported by patients and do not require hospital visits to obtain. Specifically, they include two main parts:

(1) General demographic information: including gender, age, BMI, marital status (single, married, divorced, widowed), education level (junior high school and below, high school, college, and above), and insurance coverage status (with/without insurance).

(2) Lifestyle behaviors: smoking status was classified as non-smoker, former smoker, or current smoker. Alcohol consumption status was divided into non-drinker, former drinker, or current drinker. Tea drinking status was similarly assessed as non-drinker, former drinker, or current drinker. dietary quality was measured based on the frequency of consuming midnight snacks, meat and seafood, eggs, milk and dairy products, soy products, fresh vegetables and fruits, and pickled foods. Physical activity and exercise frequency was quantified by weekly engagement levels: 5–7 days, 3–4 days, 1–2 days, ≤ 3 days per month, or never. Sleep quality was evaluated through indicators such as the presence of snoring and occurrences of insomnia. The detailed definitions of the predictive variables are provided in the Supplementary material.

### 2.5 Machine learning methods, hyperparameter optimization, and model training

In the variable selection phase, the Least Absolute Shrinkage and Selection Operator (LASSO) regression method was employed to identify the most relevant features.

Based on the selected features, nine machine learning models were developed using the training dataset: Extreme Gradient Boosting (XGBoost); Logistic Regression; Random Forest (RF); Adaptive Boosting (AdaBoost); Gaussian Naive Bayes (GNB); Complement Naive Bayes (CNB); Multilayer Perceptron (MLP); Support Vector Machine (SVM); and K-Nearest Neighbor (KNN).

**Figure 1:**
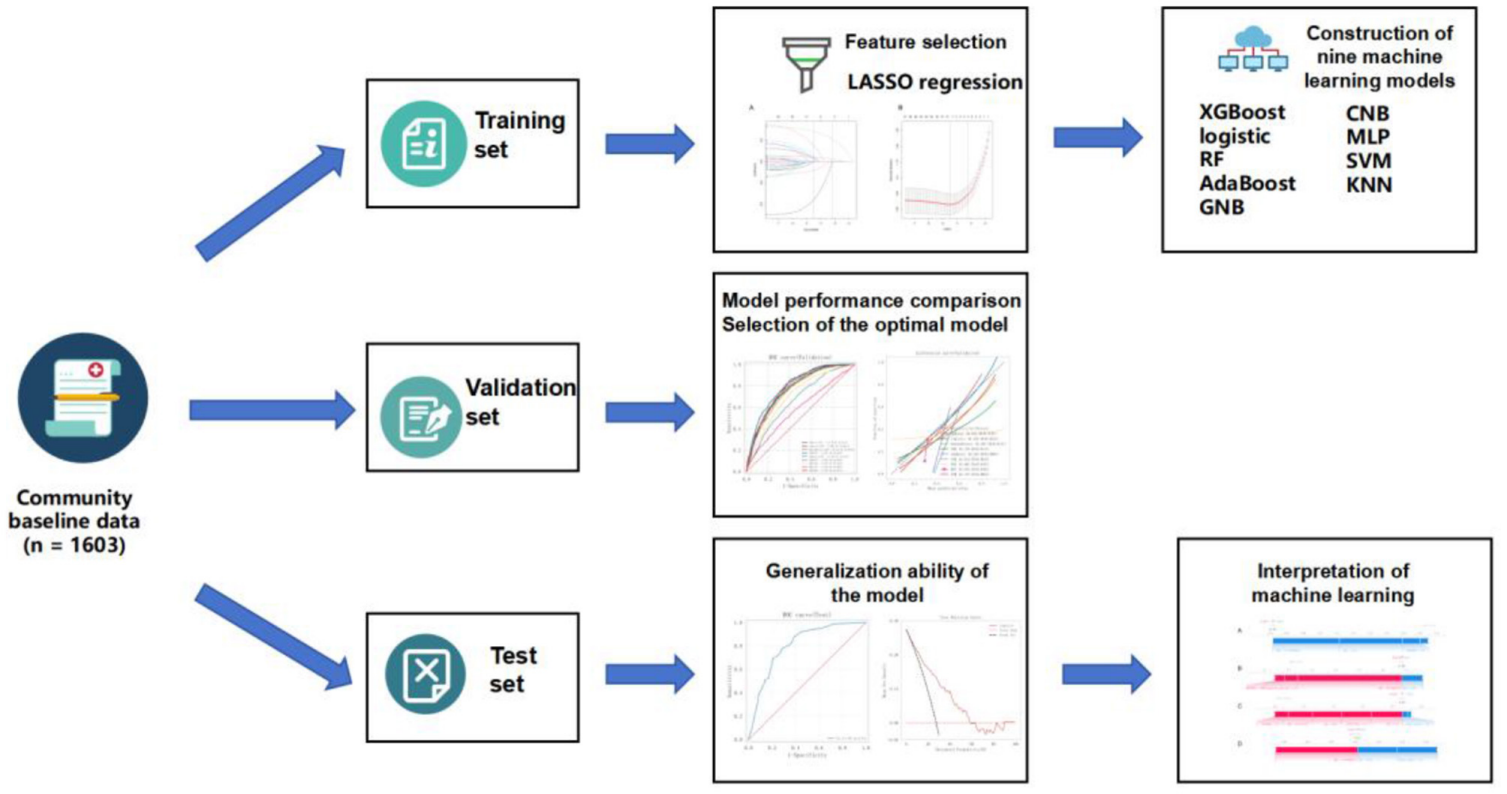
The process of machine learning models.

To optimize model performance, a systematic grid search strategy combined with resampling techniques was applied for hyperparameter tuning. The resampling process ensured that model comparisons were made under consistent data conditions, thereby improving the reliability and comparability of model evaluations. Model performance was primarily assessed using the average AUC and its variance. Once the optimal set of hyperparameters was identified, each model was retrained on the training set and subsequently evaluated on the test set to assess its generalization capability (Figure 1).

### 2.6 Model validation and evaluation

Model performance was evaluated on both the validation set and an independent test set using a comprehensive set of classification metrics, including AUC, accuracy, Cohen's Kappa coefficient, sensitivity, specificity, and F1-score. Definitions of these metrics are provided in Supplementary Table 1.

Additionally, to further assess the quality of predicted probabilities and clinical applicability, calibration curves and Decision Curve Analysis (DCA) were employed. Calibration curves evaluate the consistency between predicted probabilities and actual event rates, reflecting the model's probability calibration ability. DCA examines the net clinical benefit of a model under various decision thresholds, helping to determine its utility in real-world clinical decision-making scenarios.

### 2.7 Model interpretability analysis

To enhance interpretability, SHapley Additive exPlanations (SHAP) were applied to explain the output of the predictive models. SHAP is based on the Shapley value concept from cooperative game theory and quantifies the marginal contribution of each input variable to individual predictions. This method is model-agnostic and supports both global feature importance assessment and local interpretability for individual predictions.

## 3 Results

### 3.1 Baseline characteristics of the population

A total of 1,603 participants were included in this study, with 40% male and 60% female. The median age of the overall population was 57 years (Table 1). After a single random sampling, the participants were divided into training, validation, and test sets. The gender distribution and median age were comparable across the three subsets: the proportion of males was 41% in the training set, 37% in the validation set, and 39% in the test set; the median age in all three subsets was consistently 57 years.

**Table 1:** Basic characteristics of OSA population in Guangdong community.

**Variable**	**Total (*N* = 1,603)**	**Training (*N* = 1,025)**	**Validation (*N* = 257)**	**Test (*N* = 321)**	***P-value***
Age (years)	57.00 (48.50– 66.00)	57.00 (48.00–66.00)	57.00 (50.00– 65.00)	57.00 (49.00–65.00)	0.622
BMI, kg/m^2^	24.40 (22.26–26.59)	24.39 (22.21–26.48)	23.95 (21.88–26.43)	24.72 (22.83–27.01)	0.008
**Gender [Cases (%)]**					0.495
Male	640 (40)	420 (41)	96 (37)	124 (39)	
Female	963 (60)	605 (59)	161 (63)	197 (61)	
**MetS [Cases (%)]**					0.114
No	1,101 (69)	716 (70)	180 (70)	205 (64)	
Yes	502 (31)	309 (30)	77 (30)	116 (36)	
**Education level [Cases (%)]**					0.892
Junior high school or below	763 (48)	496 (48)	121 (47)	146 (45)	
High school	452 (28)	283 (28)	72 (28)	97 (30)	
College or above	388 (24)	246 (24)	64 (25)	78 (24)	
**Health insurance coverage [Cases (%)]**					
None	9 (0.6)	5 (0.5)	3 (1.2)	1 (0.3)	
Government-funded medical care	15 (0.9)	9 (0.9)	5 (1.9)	1 (0.3)	
Urban employee medical insurance	690 (43)	434 (42)	114 (44)	142 (44)	
Urban resident medical insurance	758 (47)	496 (48)	112 (44)	150 (47)	
New rural cooperative medical scheme	131 (8.2)	81 (7.9)	23 (8.9)	27 (8.4)	
**Smoking [Cases (%)]**					0.227
Non-smoker	1,377 (86)	892 (87)	216 (84)	269 (84)	
Former or current smoker	226 (14)	133 (13)	41 (16)	52 (16)	
**Alcohol consumption [Cases (%)]**					0.530
Non-drinker	1,337 (83)	848 (83)	220 (86)	269 (84)	
Former or current drinker	266 (17)	177 (17)	37 (14)	52 (16)	
**Tea consumption [Cases (%)]**					0.074
Non-drinker	549 (34)	368 (36)	88 (34)	93 (29)	
Former or current drinker	1,054 (66)	657 (64)	169 (66)	228 (71)	
**Midnight snack consumption [Cases (%)]**					
5–7 days per week	38 (2.4)	21 (2.0)	4 (1.6)	13 (4.0)	
3–4 days per week	25 (1.6)	15 (1.5)	6 (2.3)	4 (1.2)	
1–2 days per week	59 (3.7)	33 (3.2)	11 (4.3)	15 (4.7)	
≤ 3 days per month	123 (7.7)	82 (8.0)	16 (6.2)	25 (7.8)	
Never	1,358 (85)	874 (85)	220 (86)	264 (82)	
**Meat consumption [Cases (%)]**					0.428
5–7 days per week	1,478 (92)	940 (92)	236 (92)	302 (94)	
3–4 days per week	90 (5.6)	63 (6.1)	14 (5.4)	13 (4.0)	
1–2 days per week	22 (1.4)	12 (1.2)	7 (2.7)	3 (0.9)	
≤ 3 days per month	8 (0.5)	6 (0.6)	0 (0)	2 (0.6)	
Never	5 (0.3)	4 (0.4)	0 (0)	1 (0.3)	
**Seafood consumption [Cases (%)]**					0.939
5–7 days per week	377 (24)	241 (24)	59 (23)	77 (24)	
3–4 days per week	164 (10)	103 (10)	28 (11)	33 (10)	
1–2 days per week	446 (28)	284 (28)	78 (30)	84 (26)	
≤ 3 days per month	457 (29)	291 (28)	67 (26)	99 (31)	
Never	159 (9.9)	106 (10)	25 (9.7)	28 (8.7)	
**Egg consumption [Cases (%)]**					
5–7 days per week	587 (37)	368 (36)	91 (35)	128 (40)	
3–4 days per week	542 (34)	355 (35)	86 (33)	101 (31)	
1–2 days per week	351 (22)	223 (22)	59 (23)	69 (21)	
≤ 3 days per month	100 (6.2)	62 (6.0)	17 (6.6)	21 (6.5)	
Never	23 (1.4)	17 (1.7)	4 (1.6)	2 (0.6)	
**Milk and dairy products consumption [Cases (%)]**					0.793
5–7 days per week	210 (13)	136 (13)	34 (13)	40 (12)	
3–4 days per week	211 (13)	138 (13)	28 (11)	45 (14)	
1–2 days per week	361 (23)	217 (21)	63 (25)	81 (25)	
≤ 3 days per month	473 (30)	311 (30)	75 (29)	87 (27)	
Never	348 (22)	223 (22)	57 (22)	68 (21)	
**Soy products consumption [Cases (%)]**					0.756
5–7 days per week	57 (3.6)	37 (3.6)	8 (3.1)	12 (3.7)	
3–4 days per week	255 (16)	159 (16)	43 (17)	53 (17)	
1–2 days per week	823 (51)	535 (52)	120 (47)	168 (52)	
≤ 3 days per month	429 (27)	269 (26)	81 (32)	79 (25)	
Never	39 (2.4)	25 (2.4)	5 (1.9)	9 (2.8)	
**Vegetable consumption [Cases (%)]**					0.309
5–7 days per week	1,581 (99)	1,010 (99)	254 (99)	317 (99)	
3–4 days per week	12 (0.7)	6 (0.6)	2 (0.8)	4 (1.2)	
1–2 days per week	10 (0.6)	9 (0.9)	1 (0.4)	0 (0)	
**Fruit consumption [Cases (%)]**				
5–7 days per week	759 (47)	487 (48)	118 (46)	154 (48)	
3–4 days per week	294 (18)	187 (18)	51 (20)	56 (17)	
1–2 days per week	380 (24)	243 (24)	60 (23)	77 (24)	
≤ 3 days per month	145 (9.0)	89 (8.7)	25 (9.7)	31 (9.7)	
Never	25 (1.6)	19 (1.9)	3 (1.2)	3 (0.9)	
**Pickled products [Cases (%)]**					0.204
5–7 days per week	61 (3.8)	40 (3.9)	7 (2.7)	14 (4.4)	
3–4 days per week	102 (6.4)	69 (6.7)	11 (4.3)	22 (6.9)	
1–2 days per week	290 (18)	169 (16)	53 (21)	68 (21)	
≤ 3 days per month	890 (56)	589 (57)	141 (55)	160 (50)	
Never	260 (16)	158 (15)	45 (18)	57 (18)	
**Physical activity [Cases (%)]**					0.656
Light	1,383 (86)	892 (87)	219 (85)	272 (85)	
Moderate	167 (10)	104 (10)	27 (11)	36 (11)	
Heavy	53 (3.3)	29 (2.8)	11 (4.3)	13 (4.0)	
**Exercise [Cases (%)]**					0.893
5–7 days per week	873 (54)	567 (55)	140 (54)	166 (52)	
3–4 days per week	187 (12)	116 (11)	31 (12)	40 (12)	
1–2 days per week	169 (11)	106 (10)	29 (11)	34 (11)	
≤ 3 days per month	125 (7.8)	75 (7.3)	18 (7.0)	32 (10.0)	
Never	249 (16)	161 (16)	39 (15)	49 (15)	
**Loud snoring [Cases (%)]**					0.031
No	1,245 (78)	800 (78)	185 (72)	260 (81)	
Yes	358 (22)	225 (22)	72 (28)	61 (19)	
**Non-restorative sleep [Cases (%)]**					0.500
No	1,146 (71)	726 (71)	182 (71)	238 (74)	
Yes	457 (29)	299 (29)	75 (29)	83 (26)	
**Breathing pauses during sleep [Cases (%)]**					0.701
No	1,512 (94)	967 (94)	240 (93)	305 (95)	
Yes	91 (5.7)	58 (5.7)	17 (6.6)	16 (5.0)	
**Morning drowsiness during relaxation [Cases (%)]**					
Not drowsy	1,170 (73)	737 (72)	193 (75)	240 (75)	
Drowsy, did not fall asleep	287 (18)	193 (19)	40 (16)	54 (17)	
Drowsy, occasionally fell asleep	118 (7.4)	78 (7.6)	20 (7.8)	20 (6.2)	
Drowsy, frequently fell asleep	28 (1.7)	17 (1.7)	4 (1.6)	7 (2.2)	
**Drowsiness during inactivity [Cases (%)]**					
Not drowsy	1,319 (82)	842 (82)	208 (81)	269 (84)	
Drowsy, did not fall asleep	228 (14)	149 (15)	38 (15)	41 (13)	
Drowsy, occasionally fell asleep	43 (2.7)	27 (2.6)	10 (3.9)	6 (1.9)	
Drowsy, frequently fell asleep	13 (0.8)	7 (0.7)	1 (0.4)	5 (1.6)	
**Napping habit [Cases (%)]**					0.191
None	169 (11)	99 (9.7)	26 (10)	44 (14)	
Occasionally	287 (18)	195 (19)	41 (16)	51 (16)	
Frequently	1,147 (72)	731 (71)	190 (74)	226 (70)	
**Difficulty falling asleep [Cases (%)]**					0.892
None	741 (46)	465 (45)	119 (46)	157 (49)	
Mild	211 (13)	139 (14)	33 (13)	39 (12)	
Moderate	151 (9.4)	102 (10.0)	23 (8.9)	26 (8.1)	
Severe	106 (6.6)	67 (6.5)	21 (8.2)	18 (5.6)	
Very severe	394 (25)	252 (25)	61 (24)	81 (25)	
**Difficulty maintaining sleep [Cases (%)]**					0.752
None	847 (53)	532 (52)	141 (55)	174 (54)	
Mild	195 (12)	131 (13)	29 (11)	35 (11)	
Moderate	127 (7.9)	90 (8.8)	18 (7.0)	19 (5.9)	
Severe	97 (6.1)	62 (6.0)	16 (6.2)	19 (5.9)	
Very severe	337 (21)	210 (20)	53 (21)	74 (23)	
**Early awakening [Cases (%)]**					0.885
None	767 (48)	492 (48)	120 (47)	155 (48)	
Mild	324 (20)	200 (20)	59 (23)	65 (20)	
Moderate	149 (9.3)	99 (9.7)	20 (7.8)	30 (9.3)	
Severe	106 (6.6)	71 (6.9)	13 (5.1)	22 (6.9)	
Very severe	257 (16)	163 (16)	45 (18)	49 (15)	

Values are presented as median (interquartile range, IQR) for continuous variables and frequency (weighted percentage) for categorical variables. P-values were calculated using t-tests or Wilcoxon rank-sum tests for continuous variables and chi-square tests for categorical variables. SA, Obstructive Sleep Apnea; BMI, Body Mass Index.

Regarding the prevalence of MetS, 31% of the total population was diagnosed with MetS, with 30% in both the training and validation sets, and 36% in the test set. There were no statistically significant differences among the subsets in terms of sex, presence of MetS, educational level, smoking status, alcohol consumption, midnight snack consumption, seafood intake, vegetable intake, and various aspects of sleep status (including non-restorative sleep, breathing pauses, difficulty falling asleep, difficulty maintaining sleep, and early morning awakening). The distribution of these variables was generally consistent across the different datasets, indicating a well-balanced partitioning.

### 3.2 Variable selection

**Figure 2:**
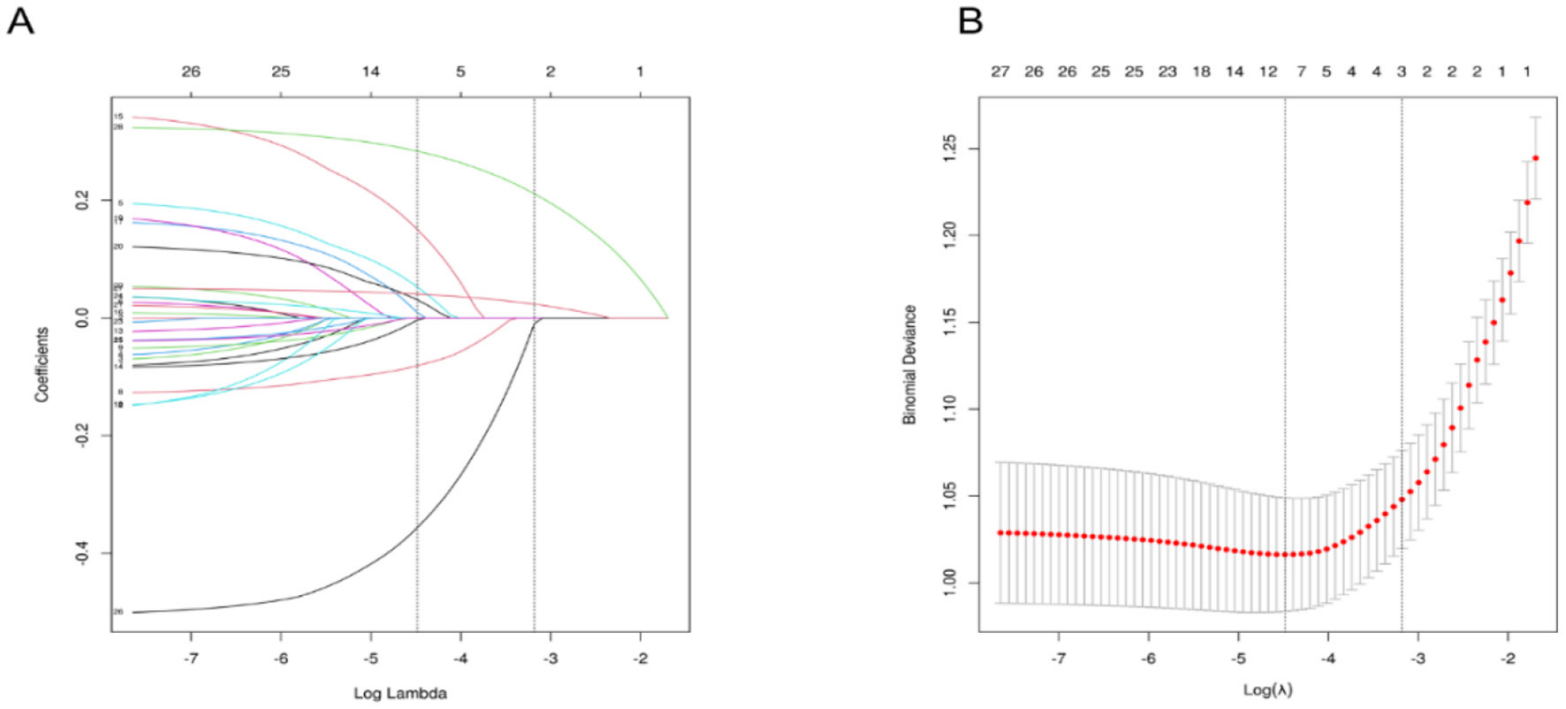
Variable Selection Using LASSO Regression. **(A)** It shows the coefficient profiles of 26 variables from the LASSO analysis. **(B)** Illustrates the selection of the optimal regularization parameter (λ) during the LASSO model fitting process, based on the minimum criteria from 10-fold cross-validation. The plot displays the relationship between binomial deviance and log(λ), with vertical dashed lines indicating the λ value that minimizes the deviance (minimum criteria) and the λ value corresponding to one standard error above the minimum (1-SE rule). LASSO, Least Absolute Shrinkage and Selection Operator.

Using LASSO regression analysis based on non-zero coefficients, nine variables were selected from the collected data, as shown in Figure 2. The selected variables included sex, age, BMI, snoring, morning sleepiness during relaxation, tea consumption, seafood intake, midnight snack consumption, and physical labor.

### 3.3 Model development and evaluation

Nine variables identified via LASSO regression were incorporated into nine distinct machine learning models. Optimal hyperparameters for each model were tuned using the validation set, as outlined in Supplementary Table 2. The performance metrics of these models on the training set were subsequently evaluated and summarized in Supplementary Table 3.

**Figure 3:**
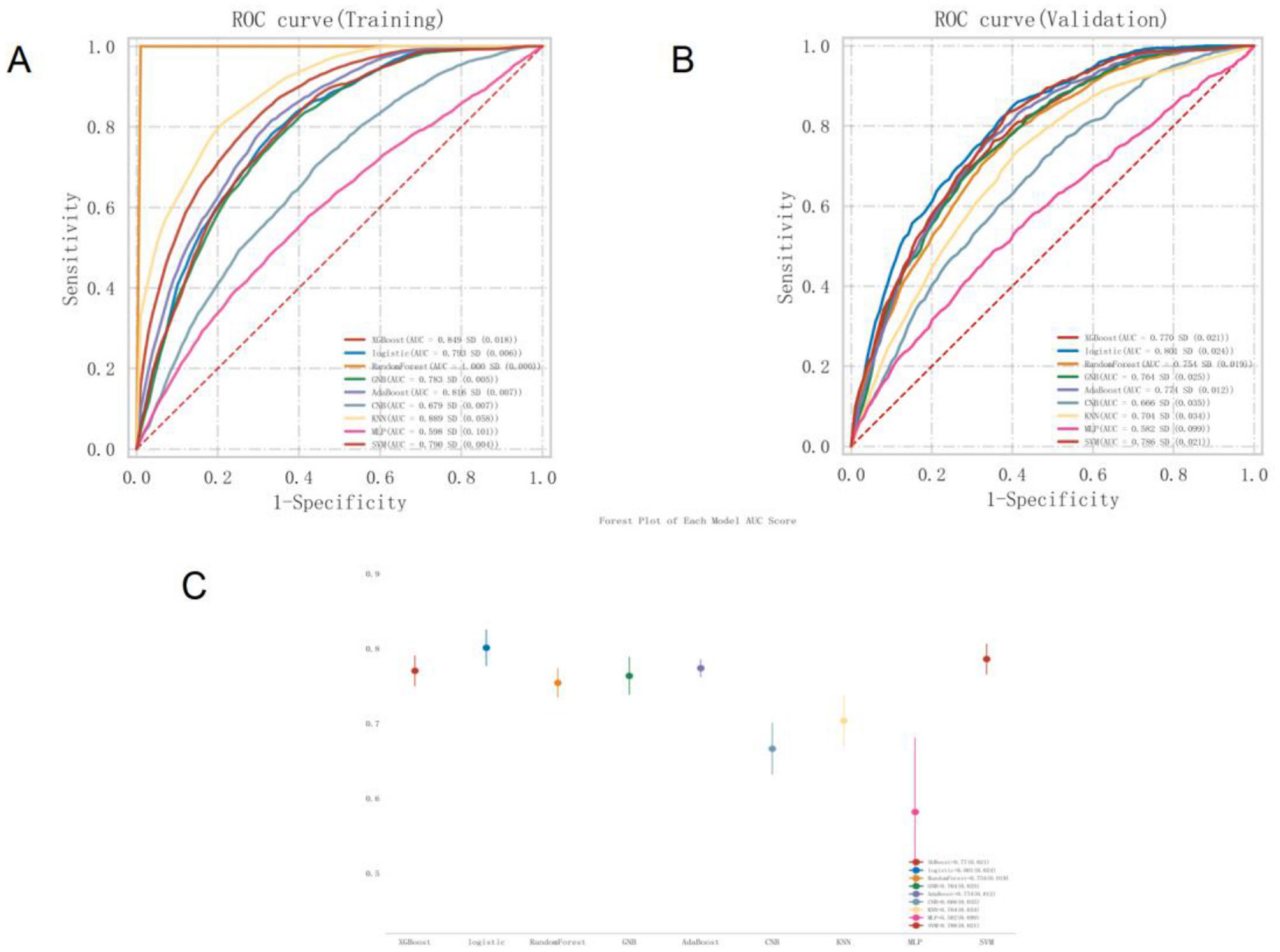
ROC curve of MetS Prediction model based on nine machine learning algorithms **(A)**. Training set **(B)**. Validation set **(C)**. AUC forest plot of MetS prediction model based on nine machine learning algorithms in validation set. ROC, Receiver Operating Characteristic; MetS, Metabolic Syndrome; AUC, Area Under the Curve.

The predictive performance of all nine models was evaluated using the validation set. The results showed that seven of the models achieved an AUC greater than 0.7. Among them, the logistic regression model demonstrated the highest AUC and the best discriminative ability. The logistic model achieved a sensitivity of 0.794, specificity of 0.647, accuracy of 0.693, F1-score of 0.617, and an AUC of 0.801, as shown in Supplementary Table 3 and Figure 3. The precision-recall (PR) curve is presented in Supplementary Figure 4. In addition, a forest plot comparing AUC scores across all nine models is shown in Figure 3C. The logistic model also performed best in calibration and decision threshold analysis, as illustrated in Supplementary Figures 5, 6. Therefore, the logistic regression algorithm was selected for further analysis.

Given that the AUC on the training set did not exceed the AUC on the validation set by more than 10%, there was no indication of significant overfitting or underfitting. This suggests that the model achieved balanced learning during training and was capable of generalizing effectively to unseen data, indicating successful model fitting (Supplementary Figure 7).

**Figure 4:**
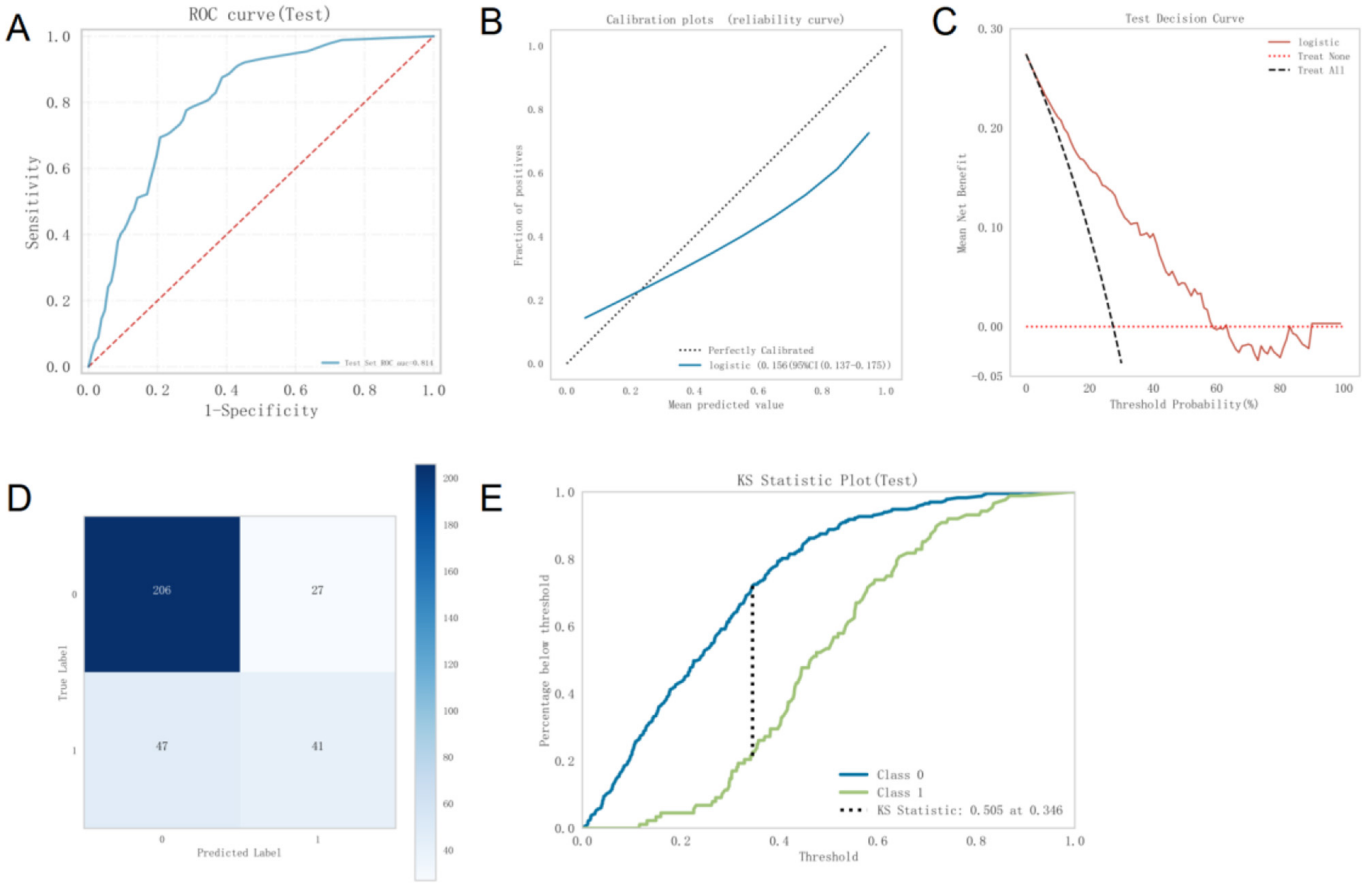
**(A)** ROC curve of the Logistic model on the test set **(B)**. Calibration curve of the Logistic model on the test set **(C)**. DCA curve of the Logistic model on the test set **(D)**. Confusion matrix of the Logistic model on the test set **(E)**. Kolmogorov-Smirnov statistic of the Logistic model. ROC, Receiver Operating Characteristic; DCA, Decision Curve Analysis; KS, Kolmogorov-Smirnov.

The best-performing logistic model on the validation set was subsequently evaluated on the test set to assess its generalizability. The model achieved an AUC of 0.814 on the test set, slightly higher than its validation AUC of 0.801, and both values exceeded 0.7, indicating strong external generalization performance (Figure 4A).

The calibration curve demonstrated strong agreement between predicted probabilities and actual outcomes (Figure 4B). DCA further showed that, within a threshold probability range of 5% to 58%, using this model to predict MetS provides significantly greater net clinical benefit compared to default strategies of treating all or no patients (Figure 4C). Thus, the model exhibits high clinical utility in moderate-risk scenarios, supporting effective decision-making and reducing unnecessary healthcare expenditures.

The confusion matrix for the test set (Figure 4D) shows that 206 instances were correctly predicted as class 0, 27 were incorrectly predicted as class 1, 47 were incorrectly predicted as class 0, and 41 were correctly predicted as class 1. The overall accuracy was calculated as (206 + 41)/(206 + 27 + 47 + 41) = 76.6%, indicating good classification performance.

The Kolmogorov–Smirnov tatistic of the model exceeded 0.23, further supporting its strong discriminative ability (Figure 4E).

### 3.4 Model interpretability using the SHAP method

**Figure 5:**
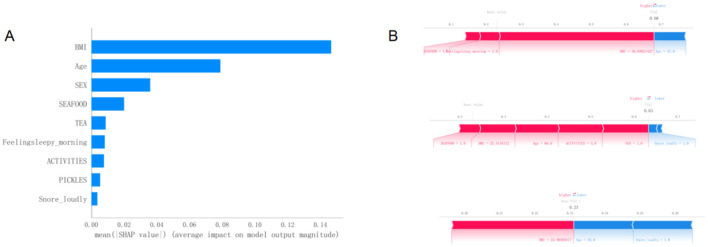
**(A)**Variable importance plot of the optimal model sorted by SHAP average values. **(B)** Visual explanation of the optimal model. SHAP, SHapley Additive exPlanations.

Figure 5A presents the SHAP summary plot of variable importance, ranked by the mean absolute SHAP values in the optimal model. The results indicate that BMI had the greatest influence on the model output, followed by age and sex. Other variables, such as seafood intake, tea consumption, morning sleepiness, physical activity, intake of pickled foods, and snoring, had relatively smaller impacts on the model's predictions.

Figure 5B provides a visual interpretation of the optimal model using SHAP bar plots to illustrate the contribution of individual variables to the model's predictions. For instance, part A of the figure demonstrates the effects of a BMI of 21.7 and an age of 41 years on the model's baseline output. A lower BMI value may indicate a reduced risk, while being 41 years old contributes to a lower estimated risk of developing MetS according to the model.

### 3.5 Web-based nomogram

A user-friendly web-based tool—an online nomogram calculator—has been developed and is freely accessible online (Supplementary Figure 8). This web calculator is specifically designed to assist community residents and healthcare professionals in estimating the probability of MetS among individuals with OSA.

### 3.6 External validation

In 2023, we collected data from 1,557 OSA patients in Guangzhou, Foshan, Qingyuan, Shenzhen, and Huazhou to perform external validation of the model. The AUC was 0.818, and model calibration also indicated acceptable fit (Supplementary Figures 9, 10).

## 4 Discussion

Risk prediction models play a pivotal role in guiding clinical and public health decision-making, with their integration into official guidelines gaining increasing prominence. In cardiovascular disease, predictive modeling has advanced significantly, exemplified by widely adopted, validated tools such as the ORISK score (19), the Framingham Risk Score (20), and the ASSIGN score (21), which are routinely utilized in clinical practice. The proliferation of large-scale datasets, combined with advancements in statistical methodologies and computational power, has further amplified the use of predictive modeling in chronic disease research. These models are essential for designing effective public health interventions to alleviate the escalating burden of chronic diseases on healthcare systems.

MetS represents a significant risk factor for a wide range of chronic conditions, including cardiovascular diseases, diabetes, and stroke, and its global prevalence is alarmingly high, with many individuals unaware of their condition. Implementing robust secondary prevention measures is critical to alleviating the substantial healthcare demands and financial costs associated with MetS. The objective of this study was to create an accessible, user-friendly self-assessment tool designed to identify individuals at elevated risk of MetS within the population diagnosed with OSA. This predictive model is distinguished to rely solely on self-reported data and straightforward, non-invasive measurements, eliminating the need for complex laboratory tests. In contrast, earlier predictive models typically relied on biomarkers such as uric acid levels, fasting plasma glucose and blood pressure readings, which necessitate specialized medical resources and professional oversight (4, 5).

The logistic regression model's superior performance in this study, despite its simplicity compared to more complex algorithms like RF, XGBoost, and SVM, warrants further discussion. Logistic regression achieved the highest AUC (0.801 in the validation set and 0.814 in the test set), indicating strong discriminative ability, and performed well in calibration and decision curve analysis. Its effectiveness can be attributed to the dataset's characteristics, where key predictors such as BMI, age, and sex exhibited strong, relatively linear associations with MetS, aligning well with logistic regression's assumption of linear relationships between predictors and the log-odds of the outcome. In contrast, complex models like RF and XGBoost, which excel at capturing non-linear relationships and higher-order interactions, may not have provided additional predictive power if such complexities were not prominent in the data. Additionally, with a moderate sample size (*n* =1,603) and a limited set of nine predictors, more complex models may have been prone to overfitting or required larger datasets to fully leverage their capabilities. Logistic regression's simplicity also enhances its interpretability, allowing clear insights into the contribution of each predictor through coefficients or SHAP values, which is critical for clinical applications where transparency is valued. For instance, the model's reliance on easily obtainable variables like BMI and age makes it practical for community-based screening. Conversely, RF and XGBoost, while potentially more accurate in datasets with complex patterns, are less interpretable due to their black-box nature, which could limit their utility in settings requiring clear decision-making rationales. SVM, effective in high-dimensional spaces, may have been less optimal due to the small number of selected features and the absence of highly non-linear boundaries in the data. Thus, logistic regression's balance of robust performance, computational efficiency, and high interpretability made it the optimal choice for this study, particularly for deployment in user-friendly tools like the web-based nomogram calculator.

### 4.1 Relationship between predictive variables and metabolic syndrome

The SHAP summary plot ranked by mean SHAP values indicated that BMI had the greatest impact on the model's output, followed by age and sex. Obesity, particularly abdominal or central obesity, is widely acknowledged as a critical risk factor for MetS. The excessive accumulation of visceral fat significantly contributes to MetS pathogenesis by stimulating the release of pro-inflammatory cytokines, notably tumor necrosis factor-alpha (TNF-α) and interleukin-6 (IL-6). These inflammatory mediators can induce insulin resistance, thereby exacerbating glucose metabolism disorders (22, 23). Additionally, obesity is closely associated with dyslipidemia which further contributes to the development of MetS (24, 25). BMI, a widely used anthropometric measure, is straightforward to calculate in everyday settings and has emerged as the most significant predictor in the MetS predictive model. Its simplicity, requiring only height and weight measurements, makes it an accessible tool for assessing MetS risk. This finding aligns with previous studies, such as Pazarl, which demonstrated that anthropometric measurements, including BMI, are strongly associated with cardiometabolic diseases in OSA patients, emphasizing their role in predicting conditions like MetS (26). Similarly, Balat et al. (27) highlighted the importance of anthropometric parameters in identifying cardiometabolic risk in OSA populations, noting their ease of measurement and clinical relevance. In a MetS prediction study based on 2,107 participants from the Isfahan cohort, support vector machine and decision tree–based models were constructed using various health characteristics, achieving sensitivities of 0.774 and 0.758, respectively (28). This study identified BMI as a key predictive variable for MetS. A supplementary analysis of 468 female participants from the same cohort further confirmed these findings, highlighting BMI as a pivotal determinant of MetS in women (29).

Age is another critical factor in the development of MetS. As individuals age, their basal metabolic rate gradually declines, leading to reduced energy expenditure and increased fat accumulation. Moreover, aging is associated with decreased insulin sensitivity, which promotes insulin resistance (30, 31). Insulin resistance interferes with normal glucose metabolism, elevating blood glucose levels and facilitating the progression of MetS. Hormonal changes related to aging, such as fluctuations in growth hormone and sex hormones, may also affect lipid and glucose metabolism (32). A comprehensive epidemiological study, drawing on data from the China Nutrition and Health Surveillance (CNHS) (2015–2017), investigated the prevalence and risk factors of Metabolic Syndrome (MetS) among Chinese adults aged 20 years and older (18). Analyzing a cohort of 130,018 participants, the study identified a significant positive correlation between advancing age and the likelihood of developing MetS. Individuals aged 45 years and above showed a markedly increased susceptibility to the condition. Statistical analysis further revealed that for each additional year of age, the risk of MetS increased by 3.7%, highlighting the progressive metabolic deterioration associated with aging.

Furthermore, the prevalence of MetS is generally higher in men than in women, which may be attributed to differences in sex hormones. Higher testosterone levels in men are associated with greater visceral fat accumulation, whereas estrogen in women promotes subcutaneous fat storage, thereby reducing visceral fat deposition (33). Increased visceral fat is closely linked to insulin resistance, hypertension, and lipid metabolism abnormalities—all core features of MetS. In addition, men are more likely to engage in unhealthy dietary and lifestyle habits, such as consuming high-calorie and high-fat foods, which further elevate the risk of MetS. A cross-sectional study conducted in the Balearic Islands among 42,146 adult workers found that the prevalence of MetS was significantly higher in men than in women. According to the ATP-III criteria, the prevalence in men was 9.4%, compared to 3.8% in women; based on the IDF criteria, the rates were 12.3% and 5.7%, respectively (34).

### 4.2 Advantages and clinical application value of the model

This study presents a predictive model comprising nine variables: BMI, sex, age, snoring, morning drowsiness, tea consumption, seafood intake, midnight snack habits, and physical labor. The model demonstrated strong discriminative ability, with an AUC of 0.793 in the training set and 0.814 in the test set. Its sensitivity, specificity, accuracy, F1 score, and AUC in the validation set were 0.794, 0.647, 0.693, 0.617, and 0.801, respectively. These results indicate that the model has good performance in identifying individuals with MetS, making it a promising tool for self-screening in community populations.

This study offers several notable advantages. First, the variables used in the model are simple, easy to obtain, and highly accessible, making it well-suited for large-scale implementation as a preliminary screening tool. By using fewer and easily collectible variables, we successfully developed an effective MetS prediction model that maintains high predictive accuracy while significantly simplifying the screening process. The model integrates multiple self-assessable indicators and eliminates the need for conventional biochemical tests or professional medical involvement, thus offering a convenient and practical tool for early detection and prevention of MetS.

From a clinical perspective, the model demonstrates strong adaptability and platform compatibility across multiple settings. For instance, hospital physical examination centers can incorporate the model into routine check-up procedures, thereby improving the accuracy and efficiency of early detection. In practice, healthcare providers can use the model's risk output to engage in in-depth discussions with patients, helping them understand individual risk factors. Particularly for individuals with OSA, the model's assessment results can support the development of personalized health management plans, enabling targeted interventions.

Moreover, the model shows distinct advantages in personalized health management. For high-risk individuals, the model can accurately identify risk factors through multidimensional profile analysis and offer tailored behavioral recommendations, such as dietary adjustments and increased physical activity. These interventions can help delay disease progression and improve individual health outcomes.

On the digital application front, the model can be fully integrated with web platforms and mobile services such as WeChat public accounts. Users can easily perform self-assessments via mobile apps or online portals. This digital approach greatly expands the model's usability and enhances public engagement, enabling OSA patients and other high-risk groups to monitor their health in real time, perform early disease risk assessments, and adopt proactive lifestyle interventions. More importantly, this self-management approach contributes to reducing the burden on healthcare systems. By encouraging individuals to take initiative in managing their health, the model helps curb the overuse of medical resources and promotes improved public health outcomes.

Finally, the model's simplicity and accessibility lend it broad social influence. Through deployment in key industries, enterprises, and at the individual level, the model facilitates early MetS risk identification across diverse populations, providing a scientific foundation for timely preventive measures. This has far-reaching strategic significance for reducing the overall prevalence of MetS and improving public health on a larger scale.

### 4.3 Limitations of this study

Despite its strengths, this study has several limitations. First, some of the variables used in the model were self-reported, which may introduce reporting bias and affect both data accuracy and model reliability. Although the study was conducted within the framework of a large-scale cohort project, with well-trained investigators and strict data quality control measures, self-reported data remain susceptible to the influence of respondents' memory, comprehension, and subjective willingness. For instance, participants may underreport or overreport their dietary intake or physical activity levels, which could impact the predictive performance of the model.

Second, the model was developed primarily based on data from a Chinese population, which may limit its external validity. Cultural background, dietary habits, and socioeconomic conditions vary significantly across countries and regions, potentially influencing the prevalence of MetS and its associated risk factors. For example, dietary patterns in China, which often include high carbohydrate intake and frequent consumption of tea or seafood, may differ from those in Western populations, where higher fat and processed food consumption is more common. Similarly, socioeconomic factors, such as access to healthcare and health literacy, can affect the prevalence of obesity and MetS risk factors, potentially reducing the model's performance in regions with different healthcare systems or economic constraints. These differences may alter the relative importance of predictive variables like BMI, tea consumption, or physical activity in other populations. To address this, future research should focus on validating the model in diverse populations, including those from Western, South Asian, and African regions, to assess its generalizability. Potential adjustments could involve recalibrating the model by incorporating region-specific variables, such as local dietary habits or socioeconomic indicators, and retraining it on datasets from diverse populations. International validation studies are planned to test the model's performance across different ethnic groups and healthcare settings, with an emphasis on adapting the model to account for cultural and environmental variations. These efforts will help ensure the model's robustness and applicability in global community settings, enhancing its utility for widespread MetS screening.

## 5 Conclusion

This study proposes a predictive model for MetS ailored to individuals with OSA in community settings. The model is based on easily accessible and straightforward variables, making it highly suitable for widespread application. As a preliminary screening tool, it requires neither biochemical tests nor professional assistance, yet can efficiently identify individuals at risk for MetS. The model has multiple potential applications in clinical practice. It can be integrated into mobile applications or online platforms, allowing individuals to conduct self-assessments and manage their health proactively. By encouraging positive lifestyle changes, the model holds promise for reducing the incidence of cardiovascular diseases and lowering healthcare expenditures.

## Data Availability

The raw data supporting the conclusions of this article will be made available by the authors, without undue reservation.
