# Supplementary material for: A predictive model for metabolic syndrome in a community-based population with sleep apnea: a secondary prevention screening tool using simple and accessible indicators
Source: Front Nutr. 2025 Nov 5;12:1667055. doi: 10.3389/fnut.2025.1667055 (PMC12626801; doi:10.3389/fnut.2025.1667055)
Supplement: Supplementary file 1 [file Table_1.docx]

**A Predictive Model for Metabolic Syndrome in a Community-Based Population With Sleep Apnea: A Secondary Prevention Screening Tool Using Simple and Accessible Indicators**

**Supplementary Table 1** Definitions of evaluation indicators

**Supplementary Table 2** Hyperparameters of nine tuned machine learning models

**Supplementary Table 3** Performance of nine machine learning models in the training set

**Supplementary Table 4** Performance of nine machine learning models in the validation set

**Supplementary Figure 1** Wearing method for the type IV wearable intelligent sleep monitor A. Front view of the sleep monitor B. Attach double-sided adhesive to the side with the photoplethysmography sensor C. The sensor is affixed to the thenar eminence region of the palm

**Supplementary Figure 2** ROC curves for diagnosing OSA using type IV sleep monitoring device

A. ROC curve for diagnosing OSA using type IV sleep monitoring device at an AHI threshold of ≥5 events/hour

B. ROC curve for diagnosing OSA using type IV sleep monitoring device at an AHI threshold of ≥15 events/hour

**Supplementary Figure 3** A. Linear correlation analysis between ODI and AHI B. Bland-Altman consistency analysis between ODI and AHI

**Supplementary Figure 4** PR curve of MetS prediction model based on nine machine learning algorithms A. Training set B. Validation set

**Supplementary Figure 5** Calibration curve plot of MetS prediction model based on nine machine learning algorithms

**Supplementary Figure 6** DCA curve plot of MetS prediction model based on nine machine learning algorithms

**Supplementary Figure 7** Fit performance of the training set and validation set under the AUC

**Supplementary Figure 8** The web-based user-friendly calculator of nomogram (https:// duanran.shinyapps.io/dynnomapp/)

**Supplementary 9** AUC of the external validation set

**Supplementary 10** Calibration curve of the external validation set

**Overall Questionnaire Design**

In this study, a standardized questionnaire was employed as the primary data collection instrument, consisting of four interrelated sections. In addition to the core content, each section included a designated investigator verification component, requiring surveyors to sign their names and provide specific remarks upon completion. This process allowed quality control personnel to promptly verify whether each section was completed according to standardized protocols, thereby ensuring data integrity.

The first section gathered sociodemographic information of the participants, including age, gender, education level, marital status, and other basic characteristics. The second section focused on the medical history of the participants and their first-degree relatives, collecting professionally diagnosed chronic disease information, such as hypertension, coronary heart disease, diabetes, and dyslipidemia. The third section captured lifestyle characteristics, including smoking history, alcohol consumption habits, and physical activity. The fourth section was dedicated to recording physiological indicators and examination results obtained during on-site physical assessments.

**Variable Definitions**

Smoking was defined as continuous smoking for more than six months; former smoking referred to previous smokers who had abstained for at least six months.

Alcohol consumption was defined as voluntary and regular drinking behavior, occurring at least twice per month, involving at least one bottle of beer or approximately 100 ml of spirits/wine per occasion. Alcohol abstinence referred to no alcohol intake for at least six consecutive months.

Physical exercise referred to sports or physical activity over the past year, with an average duration of more than 20 minutes per session. The frequency was categorized as: 5–7 days/week, 3–4 days/week, 1–2 days/week, ≤3 days/month, and never.

Occupational physical activity was classified into three levels: light, moderate, and heavy.

Light: Sedentary work with minimal movement, such as office workers, sales staff, and lecturers.

Moderate: Work involving moderate physical activity, such as vehicle drivers, electricians, lathe operators, and assembly line workers.

Heavy: Work requiring intense physical labor, such as manual farming, steelmaking, dance, professional sports, cargo handling, logging, and mining.

**Sleep Assessment**

Participants with snoring or other sleep-related issues were assessed using a specialized sleep questionnaire.

The Epworth Sleepiness Scale (ESS) is a widely used tool for evaluating daytime sleepiness. Developed by Murray W. Johns in 1991, it is particularly effective in assessing sleep disorders, especially excessive daytime sleepiness. The scale includes 8 descriptive scenarios representing different daily situations, in which participants rate their likelihood of dozing using a score from 0 to 3. The total score ranges from 0 to 24, with an ESS score ≥9 indicating the presence of daytime sleepiness.

The Insomnia Severity Index (ISI), developed by Morin and colleagues, is a standardized self-report instrument used to quantify the severity of insomnia symptoms and their impact on daily functioning. The scale includes 7 items covering aspects such as difficulty initiating sleep, maintaining sleep, early morning awakenings, sleep satisfaction, daytime impairment, concern about sleep, and overall distress. Each item is rated on a 0–4 scale (0 = no problem, 4 = very severe problem), with a total score range of 0–28. A score ≤7 indicates no insomnia, while a score >7 suggests the presence of insomnia.

**Physical Examination**

Height measurement: Conducted against a vertical wall with a level floor. Participants removed hats and shoes, standing upright with head, shoulders, buttocks, and heels touching the wall, maintaining a natural posture. The measuring device was aligned parallel to the wall and positioned accurately on the top of the head. Height was recorded to the nearest 0.1 cm.

Weight measurement: Conducted using the TANITA BC-420 Body Composition Analyzer (manufactured in Japan), placed on a flat surface with power supply connected. As the device measures body composition through foot-to-foot bioelectrical impedance, participants removed shoes and socks before measurement. After inputting the correct sex and height and selecting the appropriate mode, participants stood evenly on the measurement platform. The analyzer then displayed body weight, body fat percentage, muscle mass, and body water content. BMI (Body Mass Index) was calculated as weight (kg) divided by height squared (m²).

Supplementary Table 1 Definitions of evaluation indicators

| **Metric** | **Definition** | **Interpretation** |
| --- | --- | --- |
| **AUC** | Area under the ROC curve | Balances model performance across different thresholds; particularly suitable for imbalanced datasets. |
| **Accuracy** | Accuracy = (TP + TN) / (TP + TN + FP + FN) | A straightforward performance metric; may be biased in the presence of class imbalance. |
| **Kappa coefficient** | Kappa = (Po - Pe) / (1 - Pe) | Adjusts for class imbalance; provides a more reliable evaluation of classification performance. |
| **Sensitivity** | Sensitivity = TP / (TP + FN) | Measures the model’s ability to correctly identify positive cases; especially important when positives are rare. |
| **Specificity** | Specificity = TN / (TN + FP) | Reflects the model’s ability to correctly identify negative cases; typically evaluated alongside sensitivity. |
| **F1 Score** | F1 = 2 × (Precision × Recall) / (Precision + Recall) | Offers a balanced performance measure in imbalanced datasets; useful when false negatives carry high cost. |

Abbreviations: AUC – Area Under the ROC Curve; ROC – Receiver Operating Characteristic; TP – True Positive; TN – True Negative; FP – False Positive; FN – False Negative; Po – Observed Agreement Proportion; Pe – Expected Agreement by Chance.

Supplementary Table 2 Hyperparameters of nine tuned machine learning models

| **Model** | **AUC** | **Hyperparameters** |
| --- | --- | --- |
| **XGBoost** | 0.8489 | L2 regularization (lambda): 1; Minimum sum of instance weight (gamma): 4; Max depth: 4; Learning rate: 0.3 |
| **Logistic Regression** | 0.7931 | Convergence tolerance: 1e-6; Regularization type: L2; Max iterations: 100; Regularization strength (C): 1.0 |
| **Random Forest** | 0.9949 | Number of trees: 100; Minimum impurity decrease: 0.0; Max depth: None; Criterion: Gini |
| **Gaussian Naive Bayes (GNB)** | 0.7826 | Variance smoothing: 1e-7 |
| **AdaBoost** | 0.8154 | Number of estimators: 50; Learning rate: 0.3 |
| **Complement Naive Bayes (CNB)** | 0.6785 | Additive smoothing (Laplace/Lidstone): 0 |
| **K-Nearest Neighbors (KNN)** | 0.8874 | Weight type: Uniform; Number of neighbors: 6 |
| **Multi-Layer Perceptron (MLP)** | 0.5984 | Max iterations: 20; Hidden layer sizes: (10, 10); Activation function: Logistic |
| **Support Vector Machine (SVM)** | 0.7897 | Convergence tolerance: 0.1; Kernel type: RBF; Regularization strength (C): 1.0 |

Abbreviations: AUC – Area Under the Curve; XGBoost – Extreme Gradient Boosting; Logistic – Logistic Regression; Random Forest – Random Forest; GNB – Gaussian Naive Bayes; AdaBoost – Adaptive Boosting; CNB – Complement Naive Bayes; KNN – K-Nearest Neighbors; MLP – Multi-Layer Perceptron; SVM – Support Vector Machine.

Supplementary Table 3 Performance of nine machine learning models in the training set

| **Classification Model** | **AUC (SD)** | **Cutoff (SD)** | **Accuracy (SD)** | **Sensitivity (SD)** | **Specificity (SD)** | **Positive Predictive Value (SD)** | **Negative Predictive Value (SD)** | **F1 Score (SD)** | **Kappa (SD)** |
| --- | --- | --- | --- | --- | --- | --- | --- | --- | --- |
| XGBoost | 0.849(0.018) | 0.373(0.033) | 0.743(0.029) | 0.830(0.034) | 0.704(0.052) | 0.563(0.041) | 0.902(0.014) | 0.669(0.024) | 0.473(0.045) |
| logistic | 0.793(0.006) | 0.287(0.015) | 0.703(0.013) | 0.795(0.025) | 0.661(0.029) | 0.517(0.012) | 0.877(0.009) | 0.627(0.005) | 0.398(0.015) |
| RandomForest | 1.000(0.000) | 0.545(0.021) | 0.999(0.000) | 0.997(0.001) | 1.000(0.000) | 1.000(0.000) | 0.999(0.000) | 0.999(0.000) | 0.998(0.001) |
| GNB | 0.783(0.005) | 0.306(0.032) | 0.701(0.015) | 0.759(0.038) | 0.674(0.038) | 0.520(0.021) | 0.859(0.013) | 0.616(0.008) | 0.385(0.016) |
| AdaBoost | 0.816(0.007) | 0.484(0.008) | 0.720(0.008) | 0.807(0.030) | 0.681(0.021) | 0.536(0.010) | 0.886(0.012) | 0.644(0.010) | 0.429(0.013) |
| CNB | 0.679(0.007) | 0.483(0.010) | 0.605(0.016) | 0.712(0.052) | 0.556(0.046) | 0.426(0.015) | 0.808(0.019) | 0.532(0.009) | 0.227(0.012) |
| KNN | 0.889(0.058) | 0.533(0.245) | 0.764(0.039) | 0.462(0.259) | 0.902(0.080) | 0.558(0.058) | 0.796(0.062) | 0.646(0.112) | 0.379(0.192) |
| MLP | 0.598(0.101) | 0.430(0.334) | 0.589(0.106) | 0.594(0.234) | 0.586(0.241) | 0.487(0.122) | 0.751(0.063) | 0.573(0.147) | 0.162(0.105) |
| SVM | 0.790(0.004) | 0.281(0.030) | 0.685(0.015) | 0.821(0.043) | 0.624(0.040) | 0.497(0.016) | 0.887(0.018) | 0.618(0.006) | 0.377(0.013) |

Supplementary Table 4 Performance of nine machine learning models in the validation set

| **Classification Model** | **AUC (SD)** | **Cutoff (SD)** | **Accuracy (SD)** | **Sensitivity (SD)** | **Specificity (SD)** | **Positive Predictive Value (SD)** | **Negative Predictive Value (SD)** | **F1 Score (SD)** | **Kappa (SD)** |
| --- | --- | --- | --- | --- | --- | --- | --- | --- | --- |
| XGBoost | 0.770  (0.021) | 0.373  (0.033) | 0.686  (0.029) | 0.744  (0.071) | 0.657  (0.062) | 0.505  (0.028) | 0.849  (0.023) | 0.600  (0.031) | 0.357  (0.041) |
| logistic | 0.801  (0.024) | 0.287  (0.015) | 0.693  (0.019) | 0.794  (0.051) | 0.647  (0.031) | 0.506  (0.028) | 0.874  (0.034) | 0.617  (0.023) | 0.381  (0.034) |
| Random  Forest | 0.754  (0.019) | 0.545  (0.021) | 0.722  (0.024) | 0.408  (0.057) | 0.870  (0.042) | 0.606  (0.062) | 0.758  (0.024) | 0.483  (0.035) | 0.303  (0.037) |
| GNB | 0.764  (0.025) | 0.306  (0.032) | 0.679  (0.030) | 0.729  (0.052) | 0.657  (0.050) | 0.479  (0.038) | 0.849  (0.023) | 0.577  (0.035) | 0.336  (0.049) |
| AdaBoost | 0.774  (0.012) | 0.484  (0.008) | 0.690  (0.011) | 0.754  (0.048) | 0.660  (0.031) | 0.501  (0.022) | 0.857  (0.021) | 0.601  (0.023) | 0.364  (0.021) |
| CNB | 0.666  (0.035) | 0.483  (0.010) | 0.589  (0.033) | 0.680  (0.084) | 0.548  (0.069) | 0.397  (0.034) | 0.800  (0.032) | 0.499  (0.042) | 0.189  (0.043) |
| KNN | 0.704  (0.034) | 0.533  (0.245) | 0.689  (0.018) | 0.331  (0.214) | 0.848  (0.093) | 0.502  (0.078) | 0.748  (0.047) | 0.461  (0.065) | 0.182  (0.117) |
| MLP | 0.582  (0.099) | 0.430  (0.334) | 0.571  (0.105) | 0.581  (0.234) | 0.567  (0.243) | 0.452  (0.089) | 0.735  (0.088) | 0.174  (0.097) | 0.135  (0.097) |
| SVM | 0.786  (0.021) | 0.281  (0.030) | 0.676  (0.024) | 0.806  (0.047) | 0.615  (0.046) | 0.501  (0.032) | 0.870  (0.024) | 0.616  (0.024) | 0.362  (0.032) |


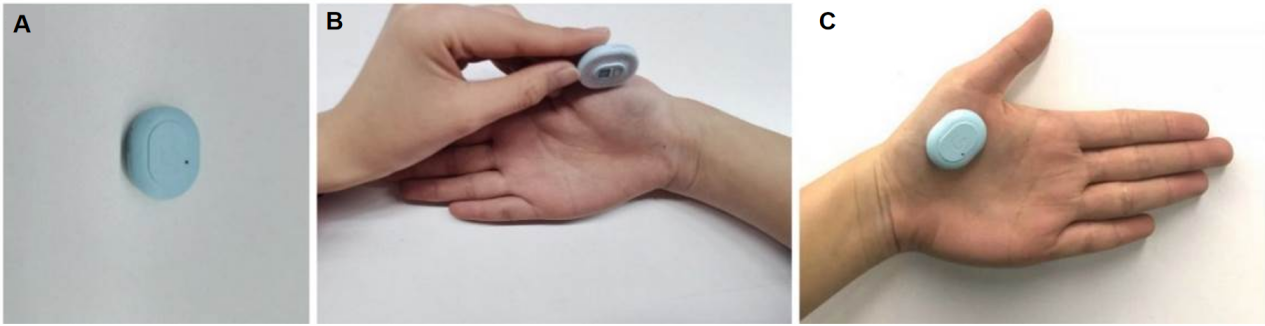


Supplementary Figure 1 Wearing method for the type IV wearable intelligent sleep monitor A. Front view of the sleep monitor B. Attach double-sided adhesive to the side with the photoplethysmography sensor C. The sensor is affixed to the thenar eminence region of the palm


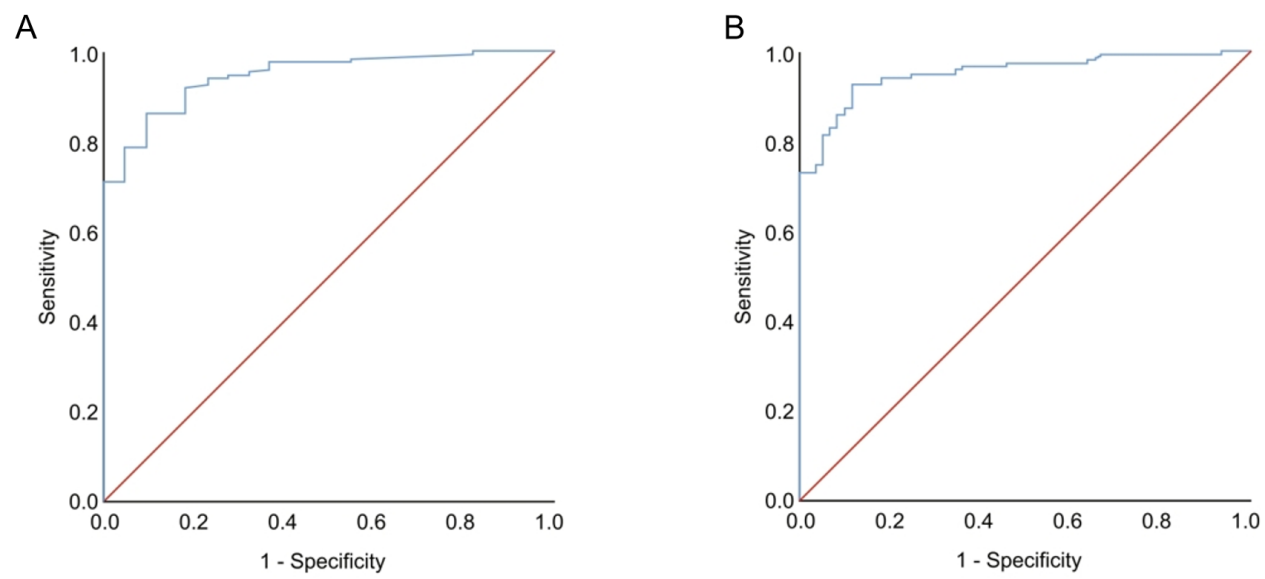


Supplementary Figure 2 ROC curves for diagnosing OSA using type IV sleep monitoring device

A. ROC curve for diagnosing OSA using type IV sleep monitoring device at an AHI threshold of ≥5 events/hour

B. ROC curve for diagnosing OSA using type IV sleep monitoring device at an AHI threshold of ≥15 events/hour


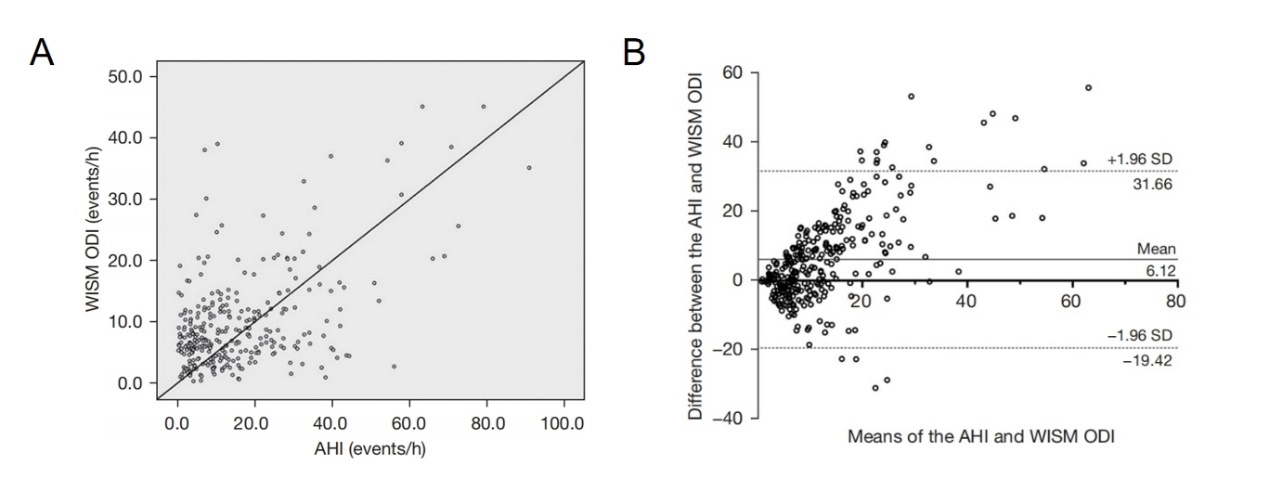


Supplementary Figure 3 A. Linear correlation analysis between ODI and AHI B. Bland-Altman consistency analysis between ODI and AHI


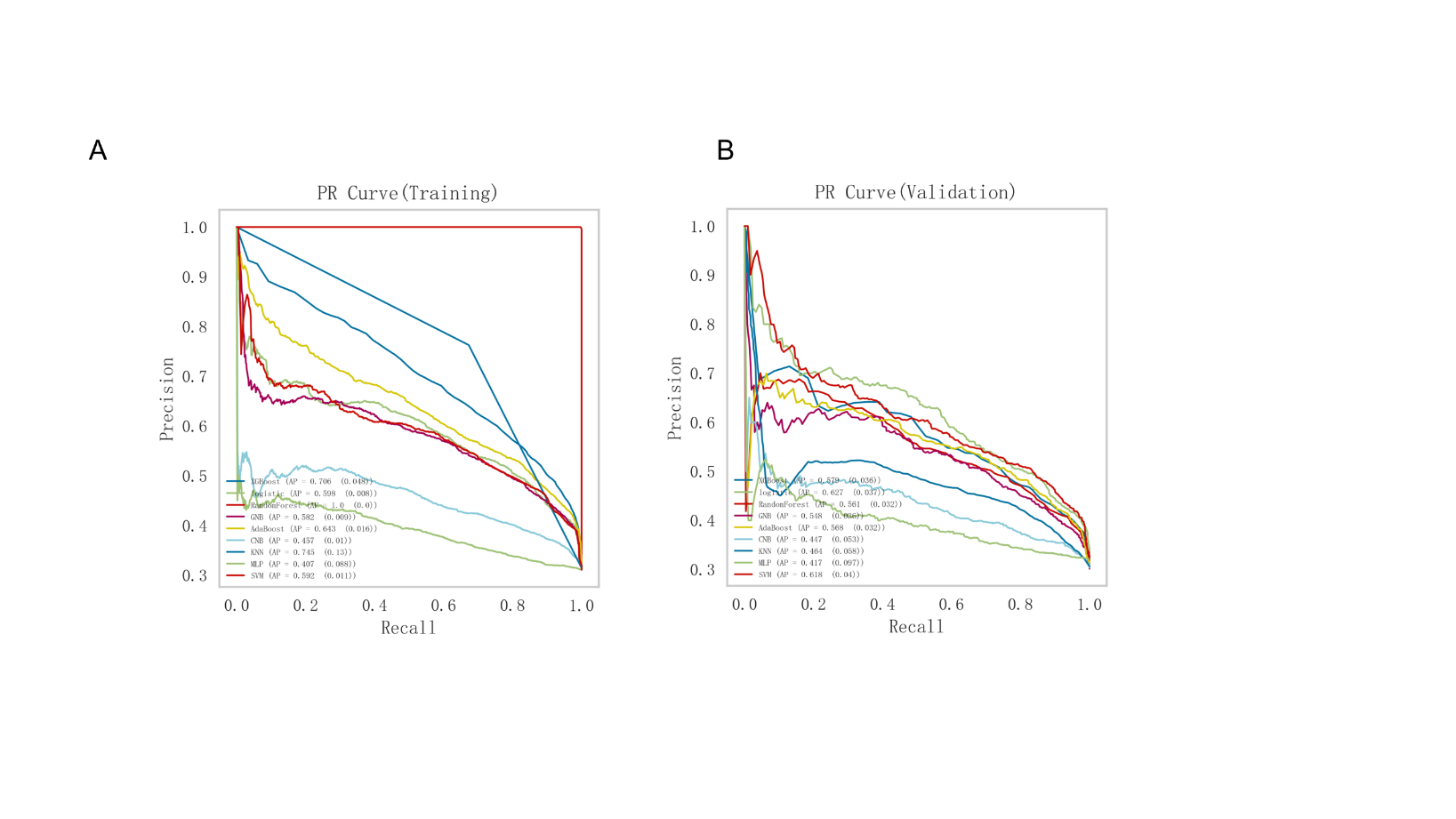


Supplementary Figure 4 PR curve of MetS prediction model based on nine machine learning algorithms A. Training set B. Validation set


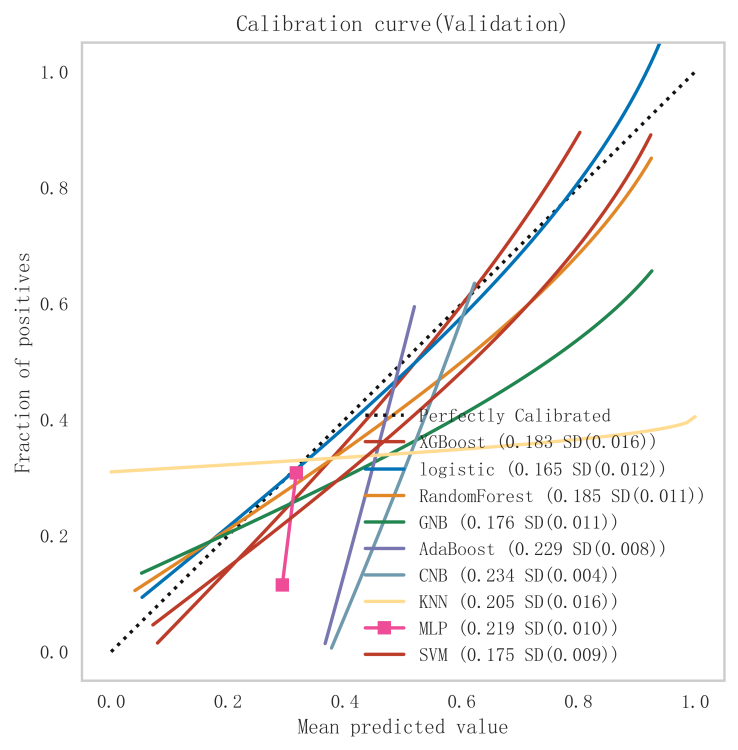


Supplementary Figure 5 Calibration curve plot of MetS prediction model based on nine machine learning algorithms


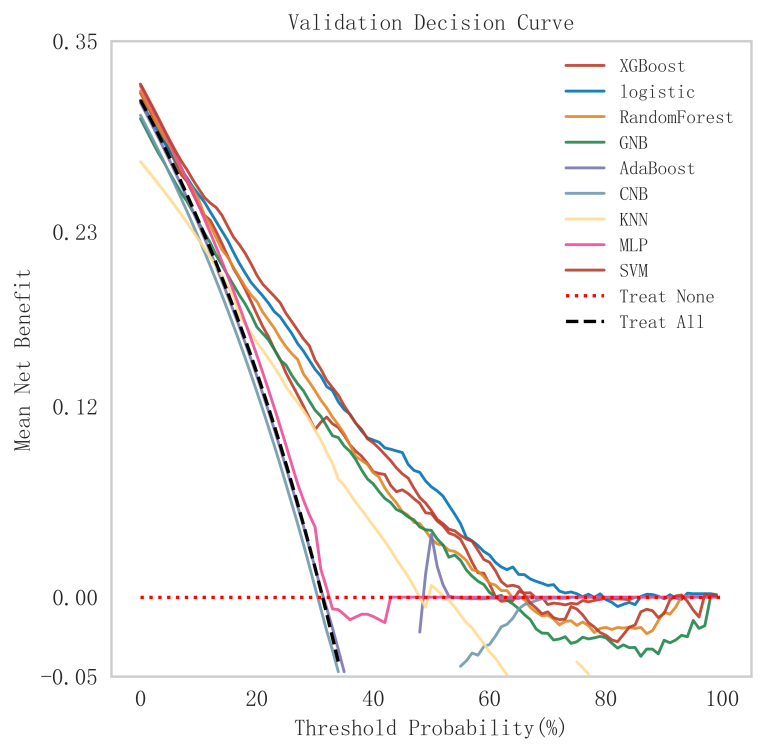


Supplementary 6 DCA curve plot of MetS prediction model based on nine machine learning algorithms


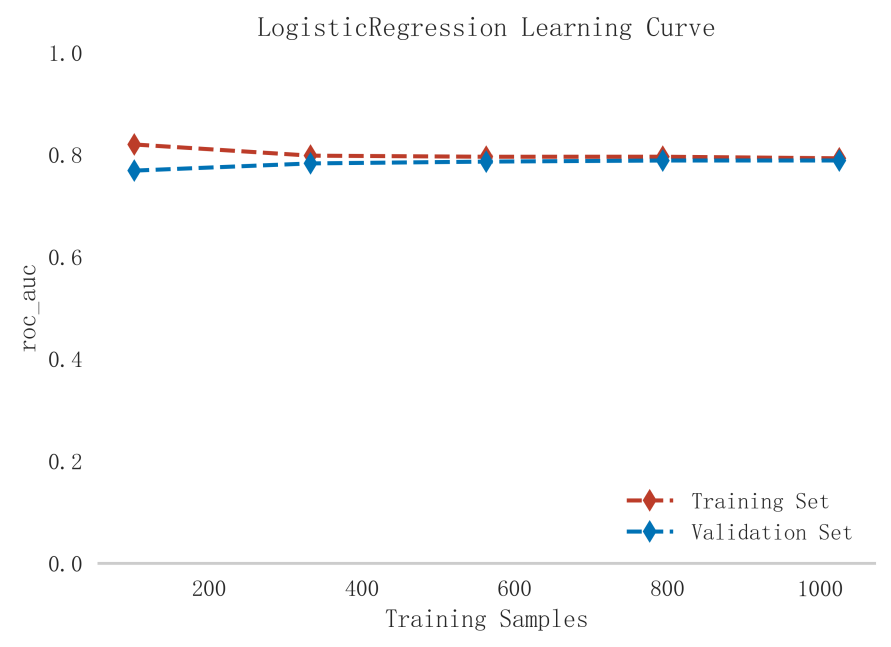


Supplementary 7 Fit performance of the training set and validation set under the AUC


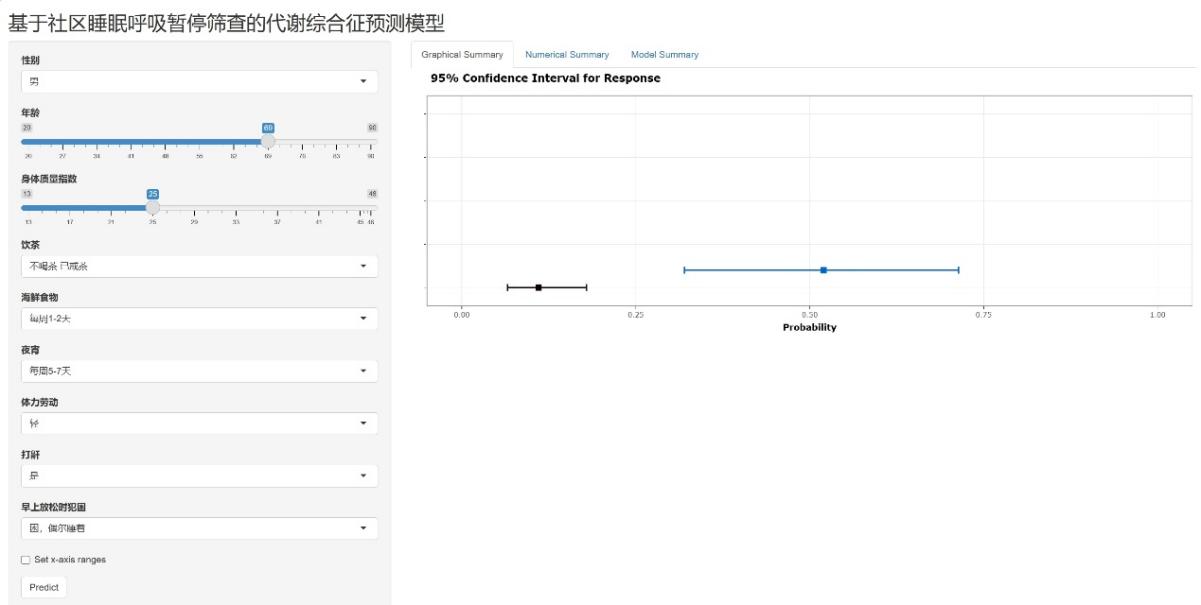


Supplementary 8 The web-based user-friendly calculator of nomogram (https:// duanran.shinyapps.io/dynnomapp/)


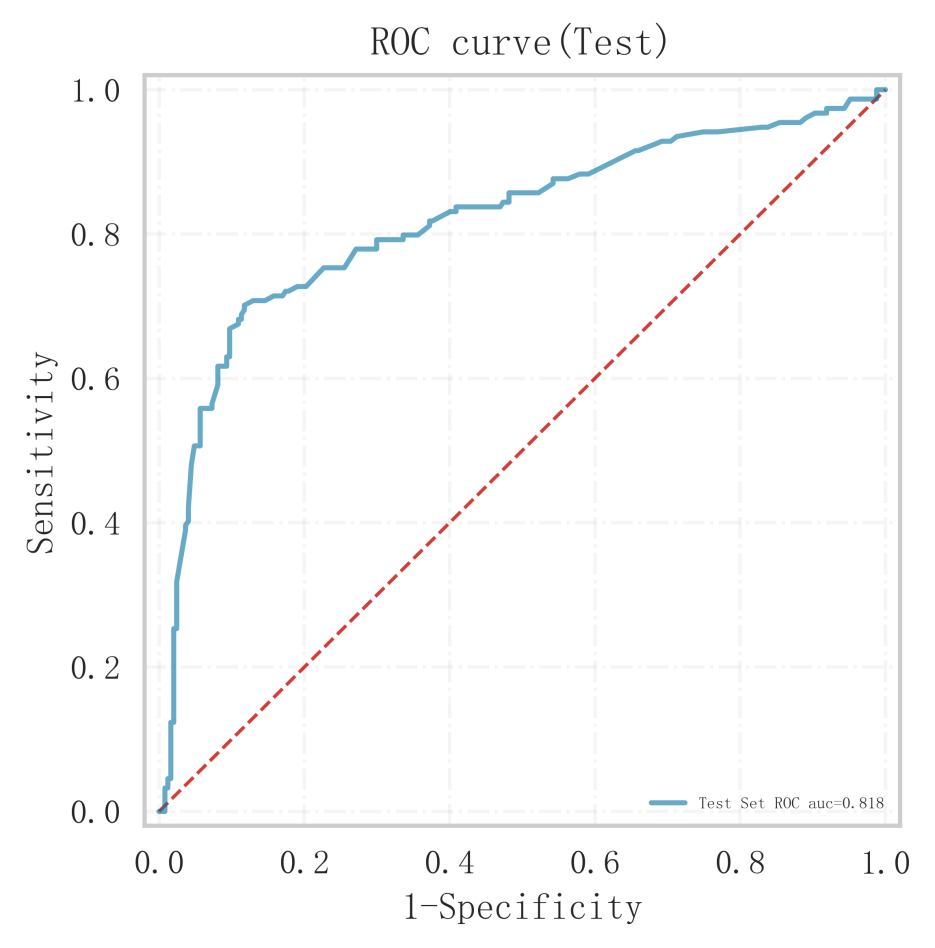


Supplementary 9 AUC of the external validation set


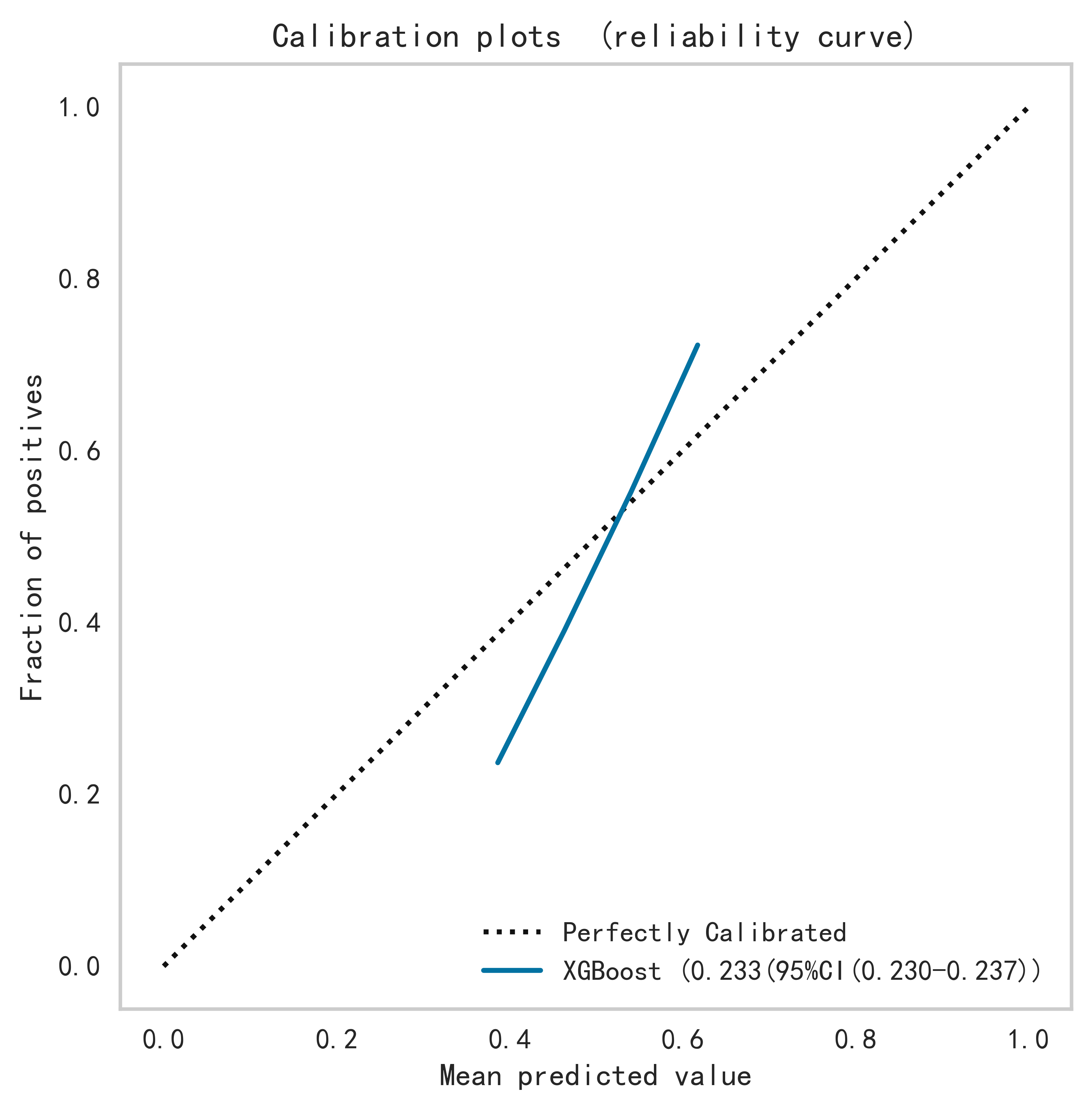


Supplementary 10 Calibration curve of the external validation set
